# Association of *N6AMT1* rs2254638 Polymorphism With Clopidogrel Response in Chinese Patients With Coronary Artery Disease

**DOI:** 10.3389/fphar.2018.01039

**Published:** 2018-09-19

**Authors:** He Li, Yan-Jiao Zhang, Mu-Peng Li, Xiao-Lei Hu, Pei-Yuan Song, Li-Ming Peng, Qi-Lin Ma, Jie Tang, Wei Zhang, Xiao-Ping Chen

**Affiliations:** ^1^Department of Clinical Pharmacology, Xiangya Hospital, Central South University, Changsha, China; ^2^Institute of Clinical Pharmacology, Central South University, Hunan Key Laboratory of Pharmacogenetics, Changsha, China; ^3^Department of Cardiovascular Medicine, Xiangya Hospital, Central South University, Changsha, China; ^4^National Clinical Research Center for Geriatric Disorders, Xiangya Hospital, Central South University, Changsha, China

**Keywords:** SNP, platelet reaction index, CAD, clopidogrel, rs2254638

## Abstract

Dual antiplatelet treatment with aspirin and clopidogrel is the standard therapy for patients undergoing percutaneous coronary intervention (PCI). However, a portion of patients suffer from clopidogrel resistance (CR) and consequently with recurrence of cardiovascular events. Genetic factors such as loss-of-function variants of *CYP2C19* contribute a lot to CR. Recently, the N-6-adenine-specific DNA methyltransferase 1 (*N6AMT1*) rs2254638 polymorphism is reported to be associated with clopidogrel response. To validate the association between *N6AMT1* rs2254638 polymorphism and clopidogrel response, 435 Chinese CAD patients receiving aspirin and clopidogrel were recruited. *N6AMT1* rs2254638 and CYP2C19^*^2/^*^3 polymorphisms were genotyped. Platelet reaction index (PRI) was measured by VASP-phosphorylation assay after treated with a 300 mg loading dose (LD) clopidogrel or 75 mg daily maintenance dose (MD) clopidogrel for at least 5 days. There was a significant difference in PRI between LD cohort and MD cohort. Carriers of CYP2C19^*^2 allele showed significantly increased PRI in the entire cohort and in respective of the MD and LD cohorts *(p* < 0.001, *p* = 0.003, *p* < 0.001, respectively). However, carriers of CYP2C19^*^3 allele exhibited significantly higher PRI only in the entire cohort and LD cohort (*p* = 0.023, *p* = 0.023 respectively). PRI value was significantly higher in *CYP2C19* PM genotyped patients as compared with those carrying the IM genotypes and EM genotype (*p* < 0.001). Besides, carriers of the rs2254638 C allele showed significantly higher PRI in entire cohort and in the LD cohort *(p* = 0.023*, p* = 0.008, respectively). When the patients were grouped into clopidogrel resistance (CR) and non-clopidogrel resistance (non-CR) groups, CYP2C19^*^2 was associated with increased risk of CR in the entire cohort, the LD cohort and the MD cohort *(p* < 0.001*, p* < 0.001, and *p* = 0.019, respectively). Carriers of the rs2254638 C allele also showed increased risk of CR in the entire cohort and the LD cohort *(p* = 0.024, and *p* = 0.028, respectively*)*. N6AMT1 rs2254638 remained as a strong predictor for CR (TC vs. TT: OR = 1.880, 95% CI = 1.099–3.216,*p* = 0.021*;* CC vs. TT: OR = 1.930, 95% CI = 1.056-3.527, *p* = 0.032; TC + CC vs. TT: OR = 1.846, 95%CI = 1.126–3.026, *p* = 0.015) after adjustment for confounding factors. Our study confirmed the influence of CYP2C19^*^2 and rs2254638 polymorphisms on clopidogrel resistance in Chinese CAD patients. Both CYP2C19^*^2 and *N6AMT1* rs2254638 polymorphism may serve as independent biomarkers to predict CR.

## Introduction

Cardiovascular disease is still the leading cause of disability and death and gives rise to tremendous financial burden worldwide. Coronary artery disease (CAD) is a common cardiovascular disease that accounts for more than half of all cardiovascular events in patients over 75 years old (Mozaffarian et al., 2015; Benjamin et al., 2018). Dual antiplatelet therapy with low dose aspirin and the P2Y12 receptor antagonist such as clopidogrel reduces risk of cardiovascular events obviously and results in the improvement in the long-term clinical outcome in CAD patients undergoing percutaneous coronary interventions (PCIs) (Tang et al., 2015; Xiao et al., 2017). However, due to neglect of inter-individual variability in clopidogrel response, ~15–40% patients suffered from clopidogrel resistance (CR) and recurrence of ischemic events during clopidogrel treatment (Amin et al., 2017).

Clopidogrel is a prodrug and is transformed into the active metabolite H4 by several cytochrome P450 (CYP450) isoenzymes. A substantial number of clinical trials have observed that CYP450 genetic polymorphisms, especially the *CYP2C19* loss-of-function (CYP2C19^*^2 and CYP2C19^*^3) and/or gain-of-function (CYP2C19^*^17) variants, can affect the antiplatelet efficacy of clopidogrel and the clinical outcomes (Chen et al., 2008a; Paré et al., 2010; Zhang et al., 2015b, 2017; Sun et al., 2016). Evidences showed that the *CYP2C19* intermediate metabolizers (IM, genotyped as CYP2C19^*^1^*^2 or CYP2C19^*^1/^*^3) and *CYP2C19* poor metabolizers (PM, genotyped as CYP2C19^*^2/^*^2, CYP2C19^*^2/^*^3, or CYP2C19^*^3^*^3) showed obviously increased risk of recurrence of ischemic events than *CYP2C19* extensive metabolizers (EM, genotyped as CYP2C19^*^1^*^1) after PCI (Mega et al., 2010; Mao et al., 2013; Zhong et al., 2018). However, only 12% of individual variability of clopidogrel response can be explained by the *CYP2C19* LOF allele, age, body mass index (BMI), and other clinical factors also contribute to this variability (Bonello et al., 2010; Jiang et al., 2015).

In a recent study, Zhong et al. identified an association of the intronic single nucleotide polymorphism (SNP) rs2254638 in N-6-adenine-specific DNA methyltransferase 1 (*N6AMT1*) with clopidogrel response in patients undergoing PCI (Zhong et al., 2017). Specially, based on genome wide association study (GWAS) in 115 coronary heart disease (CHD) patients underwent PCI, the rs2254638 variant affected both the plasma concentration of H4 and on-treatment platelet activity assessed by VerifyNow assay. Thereafter, candidate SNPs were verified in another 31 CHD patients and 32 samples of human liver S9 fraction. The authors observed that newly identified *N6AMT1* rs2254638 polymorphism was associated with clopidogrel response and H4 concentration in both pharmacokinetic replication cohort and function replication cohort. Consistent with these findings, results from an independent cohort of CHD patients suggested the rs2254638 polymorphism as a risk factor for major adverse cardiac events (MACE) after PCI (Zhong et al., 2017). Zhong's research provides further evidence for the influence of genetic variants in the pharmacokinetics pathway on clopidogrel response.

In this study, we aimed to validate the influence of CYP2C19^*^2, CYP2C19^*^3, and *N6AMT1* rs2254638 on the on-treatment platelet reactivity with clopidogrel therapy by the VASP-phosphorylation assay, and to observed whether polymorphisms in *CYP2C19* and *N6AMT1* affected clopidogrel response independently in Chinese CAD patients.

## Method

### Study subjects

CAD patients were recruited from Xiangya Hospital in Changsha, China between November 2015 and July 2017. All patients were diagnosed as CAD based on guideline of American College of Cardiology/American Heart Association (ACC/AHA). Patients were between 18 to 80 years old and were treated with clopidogrel with a loading dose of 300 mg, followed by maintenance dose of 75 mg/d. Patients with at least one of the following conditions were excluded: (a) Cardiogentic shock; (b) Intractable ventricular arrhymia; (c) NYHA grade IV congestive heart failure; (d) With a history of active internal bleeding or bleeding tendency; (e) With a severe systemic hemorrhage or hemorrhage and coagulopathy and other medical history; (f) With platelet count < 100 × 10^9^/L, INR≥1.5; (g) With hemoglobin < 10 g/dL; (h) Combination of other thienopyridine or other antiplatelet agents; (i) With severe hepatic insufficiency; (j) Merger in patients with malignant tumor. Written informed consents for genotype determination were obtained from all patients. The study was proved by the Ethics Committee of Central South University (CTXY-140002-13) and registered on the Chinese Clinical Trial Registry (ChiCTR-OPN-15006260). All protocols were performed in accordance with the Declaration of Helsinki.

### Sequencing and genotyping

Genomic DNA was extracted from peripheral blood according to the standard procedures by using a commercial DNA extraction kit (Promega, Madison, USA). DNA concentration was measured by NanoDrop 2000 spectrophotometer (NanoDrop Technologies, Wilmington, DE) and diluted into 50 ng/μl. CYP2C19^*^2 and CYP2C19^*^3 were genotyped by method of polymerase chain reaction-restriction fragment polymorphism (PCR-RFLP) (Shi et al., 2010). *N6AMT1* rs2254638 was genotyped by PCR-based sequencing by Sangon Biotechnology Company (Shanghai, China). The PCR primers were shown in Supplement Table 1.

### Platelet aggregation test

Blood samples were collected 12–24 h after taking a loading dose of clopidogrel (300 mg) or a maintenance dose of clopidogrel (75 mg/day) for at least 5 days. Blood samples were drawn into 3.8% trisodium citrate anti-coagulated vacutainer tubes and treated immediately with a commercially available VASP-phosphorylation kit (Beckman Coultronics, Margency, France) according to manufacturer's instructions. VASP-phosphorylation level was determined by flow cytometry, and PRI value was calculated.

### Statistical analysis

Continuous variables were presented as means ± SD. Comparisons between groups for continuous variables were analyzed by Mann-Whitney test or student *t*-test. Categorical variables were shown as numbers and percentages, and the comparisons between groups were analyzed by Chi-square test. Comparisons of difference in PRI among/between genotypes were carried out by Mann-Whitney test or Kruskal-Wallis test depending on the genetic models used. Patients were divided into two groups according to PRI values: clopidogrel resistant (CR) group with PRI>50%, and non-CR group with PRI ≤ 50% (Barragan et al., 2003; Bonello et al., 2008). Binomial logistic regression analysis was applied to identify the association between genotypes and CR risk after adjusting for risk factors with *p* < 0.10 in multivariable analysis. All the statistical tests were performed by using the software SPSS 19.0 (version 19.0 for Windows; Chicago, USA). A two-tailed *p* < 0.05 was deemed to statistical significance.

## Results

### Characteristics of subjects

**Figure 1:**
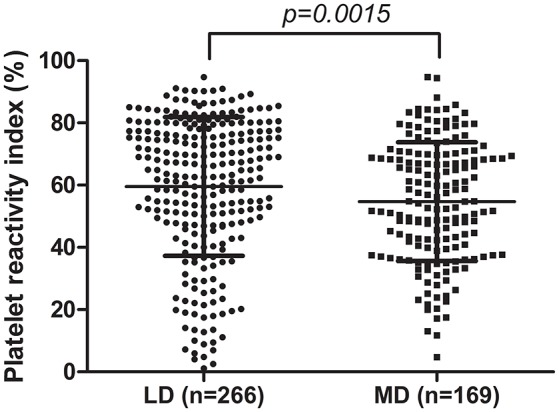
Comparison of PRI value between LD cohort and MD cohort.

A total of 435 CAD patients were recruited, the baseline characteristics of which were shown in Table 1. Briefly, there were 266 (61.1%) patients in the LD cohort and 169 (38.9%) patients in the MD cohort. The mean serum total cholesterol (TG) was significantly higher in the LD cohort as compared with the MD cohort (*p* = 0.011). More patients used calcium channel blockers (CCBs) and statins concomitantly in the MD cohort (*p* = 0.001 for CCBs, *p* < 0.001 for statins, respectively). Mean PRI value was significantly higher in the LD cohort as compared with the MD cohort (Figure 1), indicating continuous administration of clopidogrel at 75 mg daily dose for at least 5 days is more effective than a single oral dose of 300 mg, which was consistence with previous reports (Zhang et al., 2014). There was no significant difference between the LD and MD cohorts in age, sex, smoking, alcohol abuse, diabetes, hypertension, dyslipidemia, platelet count, MPV, total cholesterol, HDL-C, LDL-C, and concomitant use of proton pump inhibitors.

**Table 1:** Demographic characteristics of patients in our study.

**Characteristics**	**Entire cohort** **(*n* = 435)**	**LD cohort** **(*n* = 266)**	**MD cohort** **(*n* = 169)**	***p*^a^ value**
Age (years)	61.22 ± 9.98	60.71 ± 9.55	62.04 ± 10.59	0.178
Male, *n* (%)	279 (63.99)	178 (66.92)	101 (59.76)	0.151
Current smoke, *n* (%)	143 (32.87)	89 (33.46)	54 (31.95)	0.755
Alcohol intake, *n* (%)	95 (21.11)	61 (22.93)	34 (20.12)	0.552
Diabetes, *n* (%)	76 (21.84)	52 (19.55)	24 (14.20)	0.157
Hypertension, *n* (%)	233 (53.56)	150 (56.39)	83 (49.11)	0.141
Dyslipidemia, *n* (%)	82 (18.85)	55 (20.68)	27 (15.98)	0.258
Platelet count (10^9^/L)	206 ± 69	205 ± 68	209 ± 70	0.551
MPV (fL)	10.52 ± 2.09	10.65 ± 2.11	10.30 ± 2.06	0.113
TG (mmol/L)	2.08 ± 1.44	2.21 ± 1.56	1.85 ± 1.18	**0.011**
TC (mmol/L)	4.36 ± 1.23	4.36 ± 1.13	4.36 ± 1.39	0.957
HDL-C (mmol/L)	1.05 ± 0.28	1.05 ± 0.28	1.05 ± 0.28	0.951
LDL-C (mmol/L)	2.78 ± 1.36	2.71 ± 0.90	2.91 ± 1.89	0.229
**MEDICATION**				
PPIs, *n* (%)	208 (47.82)	119 (44.74)	89 (52.66)	0.116
CCBs, *n* (%)	82 (18.85)	37 (13.91)	45 (26.63)	**0.001**
Stains, *n* (%)	232 (53.33)	98 (36.84)	134 (79.29)	<**0.001**
PRI (%)	57.67 ± 21.22	59.56 ± 22.30	54.69 ± 19.07	**0.0015**

a *Compared between MD cohort and LD cohort; MPV, mean platelet volume; TG, triglyceride; TC, total cholesterol, HDL-C, high-density lipoprotein cholesterol; LDL-C, low-density lipoprotein cholesterol; PPIs, proton pump inhibitors; CCBs, calcium channel blockers; PRI, platelet reactivity index. Bold values mean p < 0.05*.

### Influence of the 3 SNPs on PRI during clopidogrel therapy

**Figure 2:**
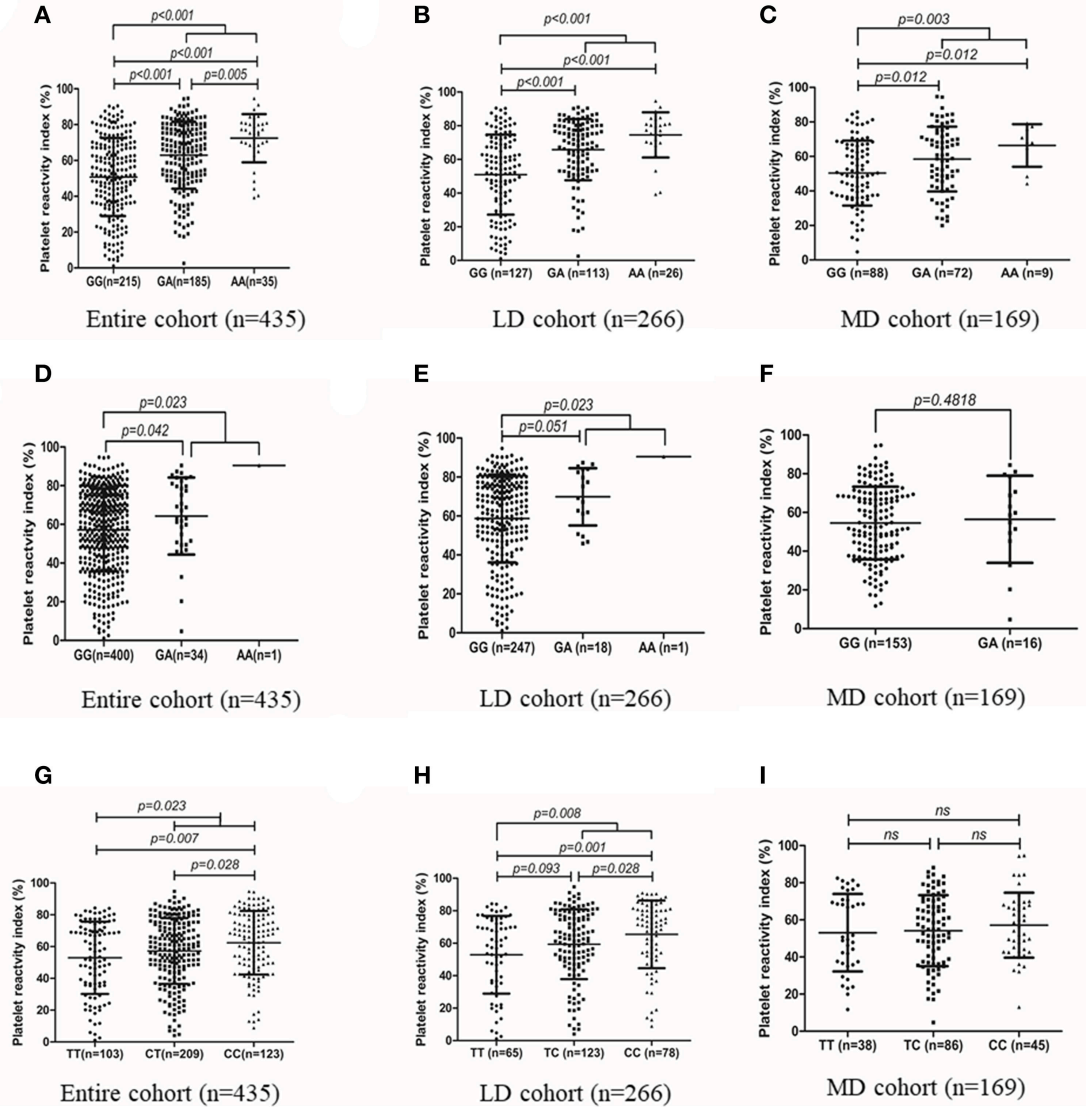
Association of CYP2C19*2 with PRI value in entire cohort **(A)**, LD cohort **(B)** and MD cohort **(C)**. Association of CYP2C19*3, with PRI value in entire cohort **(D)**, LD cohort **(E)** and MD cohort **(F)**. Association of N6AMT1 rs2254638 with PRI value in entire cohort **(G)**, LD cohort **(H)** and MD cohort **(I)**.

Genotype distribution of all the three SNPs (CYP2C19^*^2, CYP2C19^*^3, and *N6AMT1* rs2254638) were in accordance with Hardy-Weinberg equilibrium (HWE) in the patients (Supplement Table 2). Carriers of the CYP2C19^*^2 A allele showed significantly higher mean PRI than CYP2C19^*^2 GG homozygotes in the entire cohort, the LD cohort and the MD cohort, respectively (Figures 2A–C). There is a significantly difference in PRI between A allele carriers and G allele carriers of CYP2C19^*^3 in the entire cohort (64.31 ± 19.89% vs. 57.09 ± 21.25%, *p* = 0.023) (Figure 2D). In LD cohort, CYP2C19^*^3 A allele carriers showed significantly higher PRI value than those with the CYP2C19^*^3 GG homozygotes (70.92 ± 15.03% vs. 58.69 ± 22.55*%, p* = 0.023) (Figure 2E). As compared with the *N6AMT1* rs2254638 CC homozygotes, rs2254638 T allele carriers exhibited significantly lower PRI in both the entire cohort and the LD cohort (Figures 2G,H). Neither CYP2C19^*^3 nor *N6AMT1* rs2254638 polymorphisms showed effect on PRI in the MD cohort (Figures 2F,I).

**Figure 3:**
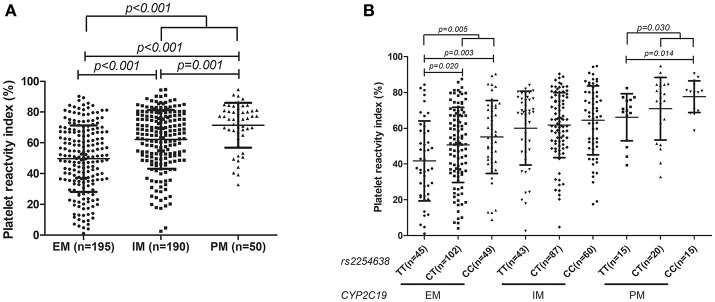
Association of distribution of *CYP2C19* metabolic phenotype with PRI **(A)** and the influence of rs2254638 polymorphism on PRI in different *CYP2C19* metabolic phenotype **(B)**.

Stratification analysis for influence of *N6AMT1* rs2254638 on PRI based on the *CYP2C19* metabolic phenotype was further carried out. We observed that *CYP2C19* PM patients showed significantly higher PRI than IM and EM patients, respectively, and a gene-dose effect was observed (Figure 3A). The influence of rs2254638 polymorphism on PRI was observed in both *CYP2C19* EM patients and PM patients, but not in *CYP2C19* IM patients (Figure 3B).

### Association of clinical characteristics with CR in the CAD patients

**Figure 4:**
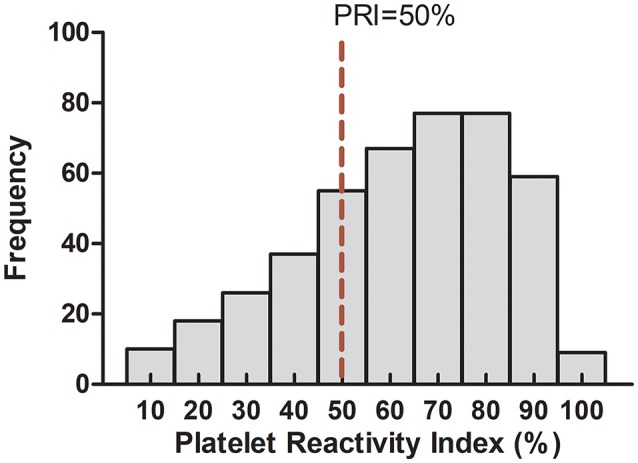
Detailed frequency distribution of the study subjects in relation to platelet reactivity index.

In our study, the PRI values ranged from 1 to 95% (mean 57.67 ± 21.22) and were in accordance with a normal distribution (Figure 4). When the cut-off value was identified as 50% according to previous studies (Barragan et al., 2003; Blindt et al., 2007; Bonello et al., 2007), 289 (64.22%) of the patients were identified as CR, 166 (35.78%) of the patients were identified as non-CR. The presence of hypertension, high level of TG and LDH-C significantly increased the risk of CR. However, concomitant use of statins significantly decreased the risk of CR, which was consist with the previous reports which indicated that high dose atorvastatin significantly enhanced clopidogrel efficacy possibly through affecting clopidogrel bioactivation (Farid et al., 2008; Leoncini et al., 2014). Moreover, patients with dyslipidemia had a tendency to increase the risk of CR (*p* = 0.068). There was no statistical difference with respect to other clinical characteristics between the CR and non-CR groups (Table 2).

**Table 2:** Risk prediction of clinical characteristics for CR in our study.

**Risk factors**	**CR** **(*n* = 289)**	**Non-CR** **(*n* = 146)**	**OR (95% CI)**	***p* value**
Age (years)	60.71 ± 9.79	62.25 ± 10.30	0.984 (0.965–1.005)	0.130
Male, n (%)	185 (64.01)	94 (64.38)	1.016 (0.671–1.539)	0.940
Current smoke, n (%)	96 (33.22)	47 (32.19)	1.048 (0.685–1.602)	0.829
Alcohol intake, n (%)	63 (21.80)	32 (21.92)	0.993 (0.614–1.607)	0.99
Diabetes, n (%)	49 (16.96)	27 (18.49)	0.900 (0.536–1.511)	0.714
Hypertension, n (%)	143 (49.48)	56 (38.36)	1.614 (1.094–2.461)	0.019^*^
Dyslipidemia, n (%)	61 (21.11)	21 (14.38)	1.593 (0.926–2.738)	0.068
Platelet count (10^9^/L)	204 ± 65	212 ± 76	0.998 (0.995–1.001)	0.274
MPV (fL)	10.56 ± 2.16	10.45 ± 1.97	1.027 (0.929–1.134)	0.607
TG (mmol/L)	2.24 ± 1.54	1.19 ± 1.19	1.307 (1.087–1.572)	0.002^*^
TC (mmol/L)	4.47 ± 1.18	4.15 ± 1.28	0.673 (0.336–1.347)	0.351
HDL-C (mmol/L)	1.05 ± 0.28	1.06 ± 0.27	0.890 (0.420–1.885)	0.761
LDL-C (mmol/L)	2.92 ± 1.48	2.54 ± 1.07	1.421 (1.118–1.806)	0.009^*^
**MEDICATION**				
**Clopidogrel**
75 mg ≥ 5 d, *n* (%)	97 (57.40)	72 (42.60)	1.926 (1.283–2.891)	0.001^*^
PPIs, *n* (%)	130 (44.98)	78 (53.42)	0.713 (0.478–1.063)	0.096
CCBs, *n* (%)	51 (17.65)	31 (21.23)	1.258 (0.764–2.072)	0.368
Stains, *n* (%)	144 (49.83)	91 (61.07)	0.627 (0.419–0.940)	0.016^*^
PRI (%)	70.15 ± 11.28	32.96 ± 13.00	1.749 (1.396–2.192)	<0.001^*^

* *Means p < 0.05; MPV, mean platelet volume; TG, triglyceride; TC, total cholesterol, HDL-C, high-density lipoprotein cholesterol; LDL-C, low-density lipoprotein cholesterol; PPIs, proton pump inhibitors; CCBs, calcium channel blockers; PRI, platelet reactivity index. Bold values mean p < 0.05*.

### Associations of the three SNPs with risk of CR in CAD patients

In the entire cohort, CYP2C19^*^2 was associated with increased risk of CR in co-dominant, dominant and recessive models (*p* < 0.001, *p* < 0.001, and *p* = 0.004, respectively). *N6AMT1* rs2254638 was also associated with CR risk in dominant model for entire cohort (*p* = 0.024) (Table 3). In the LD cohort and MD cohort, the distribution of CYP2C19^*^2 was statistically significant between CR group and non-CR group (*p* < 0.001 and *p* = 0.0019, respectively) (Table 4). The presence of rs2254638 C allele showed increased risk of CR in comparison to the wild type genotype in the LD cohort (*p* = 0.028). Besides, rs2254638 showed marginally increased risk of CR in co-dominant model between CR and non-CR groups in entire cohort and LD cohort population (*p* = 0.063, *p* = 0.054, respectively). However, CYP2C19^*^3 had no predictive value to CR in all cohorts and all analysis models (Tables 3, 4).

**Table 3:** Allelic frequencies of all gene between patients with or without clopidogrel resistance in entire cohort.

**Gene/SNP**	**Genotype**	**CR** **(*n* = 289)**	**Non-CR** **(*n* = 146)**	**Co-dominant** ***p* value**	**Dominant** ***p* value**	**Recessive** ***p* value**
CYP2C19*2	GG	115 (39.79)	100 (68.49)	<**0.001**	<**0.001**	**0.004**
	GA	143 (49.48)	42 (28.77)			
	AA	31 (10.73)	4 (2.74)			
CYP2C19*3	GG	262 (90.66)	138 (94.52)	0.333	0.162	0.477
	GA	26 (9.00)	8 (5.48)			
	AA	1 (0.34)	0 (0)			
N6AMT1 rs2254638	TT	59(20.42)	44(30.14)	**0.063**	**0.024**	0.157
	CT	142 (49.13)	67 (45.89)			
	CC	88 (30.45)	35(23.97)			

*Bold values mean p < 0.05*.

**Table 4:** The association between the 3 SNPs and clopidogrel resistance in LD cohort and MD cohort.

**Gene/SNP**	**Genotype**	**LD (*****n*** = **266)**					**MD (*****n*** = **169)**				
		**CR** **(*n* = 192)**	**Non-CR** **(*n* = 74)**	**Co-dominant** ***p* value**	**Dominant** ***p* value**	**Recessive** ***p* value**	**CR** **(*n* = 97)**	**Non-CR** **(*n* = 72)**	**Co-dominant** ***p* value**	**Dominant** ***p* value**	**Recessive** ***p* value**
CYP2C19^*^2	GG	72 (37.50)	55 (74.32)	<**0.001**	<**0.001**	**0.016**	43 (44.33)	45 (62.50)	**0.050**	**0.019**	0.204
	GA	96 (50.00)	17 (22.97)				47 (48.45)	25 (34.72)			
	AA	24 (12.50)	2 (2.71)				7 (7.22)	2 (2.78)			
CYP2C19^*^3	GG	176 (91.67)	71 (95.95)	0.448	0.225	—	86 (88.66)	67 (93.06)	0.334	0.334	—
	GA	15 (7.8)	3 (4.05)				11 (11.34)	5 (6.94)			
	AA	1 (0.52)	0 (0)				—	—			
N6AMT1	TT	40 (20.83)	25 (33.78)	**0.054**	**0.028**	0.087	19 (19.59)	19 (26.39)	0.553	0295	0.952
rs2254638	CT	90 (46.88)	33 (44.59)				52 (53.61)	34 (47.22)			
	CC	62 (32.29)	16 (21.62)				26 (26.80)	19 (26.39)			

*Bold values mean p < 0.05*.

Unconditional logistic regression was further performed after adjusting for the covariates potentially affect PRI. The results showed that the CYP2C19^*^2 was a major risk factor for CR (GG vs. GA: OR: 3.157, 95% CI: 1.981–5.031, *p* < 0.001; GG vs. AA: OR: 7.641, 95% CI: 2.496-23.394, *p* < 0.001; GG vs. GA + AA: OR: 3.526, 95% CI: 2.244–5.541*, p* < 0.001; GG +GA vs. AA: OR: 4.708, 95% CI: 1.591–13.926, *p* = 0.005*). N6AMT1* rs2254638 remained an independent risk factor for CR (TT vs. TC: OR: 1.880, 95% CI: 1.099–3.216*, p* = 0.021; TT vs. CC: OR: 1.930, 95% CI: 1.056–3.527, *p* = 0.032; TT vs. TC + CC: OR: 1.846, 95%CI: 1.126–3.026*, p* = 0.015, Table 5).

**Table 5:** Association of 3 SNPs with CR in 435 patients by binomial logistic regression analysis.

**Gene/SNP**	**Genotype**	**Non-CR**	**CR**	**OR^a^ (95%CI)**	***p*^a^ Value**
CYP2C19*2	GG (%)	115 (39.79)	100 (68.49)	1.0 (ref)	
	GA (%)	143 (49.48)	42 (28.77)	3.157 (1.981–5.031)	**0.000**
	AA (%)	31 (10.73)	4 (2.74)	7.641 (2.496–23.394)	**0.000**
	GG vs. GA+AA			3.526 (2.244–5.541)	**0.000**
	GG+GA vs. AA			4.708 (1.591–13.926)	**0.005**
CYP2C19*3	GG (%)	262 (90.66)	138 (94.52)	1.0 (ref)	
	GA (%)	26 (9.00)	8 (5.48)	2.356 (0.977–5.679)	0.056
	AA (%)	1 (0.34)	0 (0)		
	GG vs. GA+AA			2.168 (0.920–5.108)	0.077
	GG+GA vs. AA				
N6AMT1 rs2254638	TT (%)	59(20.42)	44(30.14)	1.0 (ref)	
	TC (%)	142 (49.13)	67 (45.89)	1.880 (1.099–3.216)	**0.021**
	CC (%)	88 (30.45)	35(23.97)	1.930 (1.056–3.527)	**0.032**
	TT vs. TC+CC			1.846 (1.126–3.026)	**0.015**
	TT+TC vs. CC			1.296 (0.797–2.109)	0.296

OR, odds ratio; CI, confidence interval;

a *Means adjusting variables include age, smoking, alcohol intake, hypertension, diabetes, Dyslipidemia, total cholesterol, low-density lipoprotein cholesterol, proton pump inhibitors, calcium channel blockers, stains, and clopidogrel dose. Bold values mean p < 0.05*.

## Discussion

A growing body of evidence has suggested the critical role of *CYP2C19* in clopidogrel bioactivation. It is widely acknowledged that the loss of function variations in *CYP2C19* (CYP2C19^*^2 and CYP2C19^*^3) affect clopidogrel response and clinical outcome (Hulot et al., 2006; Mega et al., 2009; Shuldiner et al., 2009). Both CYP2C19^*^2 and CYP2C19^*^3 lead to truncated proteins by producing early-stop codons (Cui et al., 2015). Recently, a GWAS suggested that *N6AMT1* rs2254638 polymorphism was associated with P2Y12 reaction unit (PRU) and H4 concentration *ex vivo* in CAD patients with clopidogrel therapy (Zhong et al., 2017). Furthermore, the polymorphism was identified to exert a marginal risk effect for MACE in an independent patient cohort (Zhong et al., 2017).

In our study, 435 CAD Chinese patients were enrolled to analyze the influence of CYP2C19^*^2, CYP2C19^*^3, and *N6AMT1* rs2254638 on clopidogrel response by VASP-PRI assay. Consistent with the most of previous findings, we observed that carriers of the CYP2C19^*^2 allele showed significantly higher PRI and increased risk of clopidogrel resistance, which further support the predictive role of this polymorphism on clopidogrel efficacy in Chinese population. Furthermore, we observed that carriers of the rs2254638 C allele demonstrated significantly higher PRI and increased risk of clopidogrel resistance as compared with rs2254638 TT homozygote genotype in the entire patient cohort and the LD patient cohort. Though no difference between *N6AMT1* rs2254638 C allele carriers and TT homozygotes was observed in the MD cohort (*p* = 0.556), rs2254638 C carriers also showed a trend of increased PRI (TT vs. TC vs. CC: 53.08 ± 20.85%; 54.13 ± 19.13%; 57.12 ± 17.54%). In addition, the difference in PRI among N6AMT1 rs2254638 genotypes was observed in both CYP2C19 EM and PM genotyped patients. Recently, both *ex vivo* studies and clinical outcome studies have evaluated the influence of environment factors, especially co-administration drugs to the clopidogrel response (Jiang et al., 2015; Amin et al., 2017). Previous evidences assessed the interaction of SNPs and environment factors through conducting multivariable logistic regression models (Hung et al., 2004; Zollanvari and Alterovitz, 2017). By using binomial logistic regression analysis to adjust the influence of environment factor on clopidogrel response, our findings indicated that CYP2C19^*^2 and N6AMT1 rs2254638 polymorphisms were still independent genetic predictive factors for CR in Chinese population. However, CYP2C19^*^3 alone affected PRI value only in the entire cohort and LD cohort, and no association between CYP2C19^*^3 polymorphism and CR was observed in our study. As shown in our results, only 1 CYP2C19^*^3/^*^3 homozygote was observed in our samples, and the frequency of CYP2C19^*^3 is extremely low in Chinese population (3.3% according to literature report) (Chen et al., 2008b). These may limit the possibility of obtaining a statistically significant association with sufficient power of accuracy with our sample size.

*N6AMT1* encodes for N (6)-adenine-specific DNA methyltransferase. There are few studies focused on function of N6AMT1 previously. The enzyme may be involved in the methylation of release factor I (Liu et al., 2010). Besides, it is involved in converting the arsenic metabolite monomethylarsonous acid to the lesstoix dimethylarsonic acid (Ren et al., 2011; Zhang et al., 2015a). N6AMT1 polymorphisms are reported to be associated with arsenic methylation (Harari et al., 2013; Chen et al., 2017; de la Rosa et al., 2017). N6AMT1 rs2254638 is a T to C substitution in intron, and the exact function of the SNP remains unknown. By using the 1000 genome database and Haploview software, we observed that 7 SNPs including rs2243615, rs2254507, rs2738963, rs2103490, rs2251224, rs1987787, and rs1987786 were in strong linkage disequilibrium with rs2254638 (r2 > 0.8, data not shown). Especially, rs1987787 and rs1987786 were located in the predicted binding sites of miRNAs (www.snpinfo.niehs.nih.gov). The N6AMT1 rs2254638 polymorphism was also presumed to be related to the N6AMT1 expression in multi-tissues as indicated by the eQTL database (www.gtexportal.org). We assumed that N6AMT1 rs2254638 or SNPs in LD with the polymorphism may act through regulating N6AMT1 expression and its activity consequently. The study by Zhong et al observed that the N6AMT1 rs2254638 can influence clopidogrel efficacy through affecting its metabolism, but the exact mechanisms deserved further study.

Several methods of measuring platelet aggregation *ex vivo* have been developed (Breet et al., 2010). However, most methods evaluate the comprehensive influence of ADP-induced platelet aggregation through both P2Y12 and P2Y1 receptors. VASP-PRI assay is the only measurement that can exclude the contribution of P2Y1 receptor to the overall response (Cuisset et al., 2009). What's more, the VASP-PRI analysis can correctly evaluate the clopidogrel efficacy to LD prior PCIs, which perfectly meets our need (Bonello et al., 2007, 2008, 2009; Cuisset et al., 2009). Two limitations merit mention in our study. The first is that we could not preclude the false possibility of type II error due to the small sample size for the MD cohort. Secondly, the short-term outcome and long-term clinical outcome is missing, which should be replenished in our future studies.

In conclusion, we confirmed the influence of CYP2C19^*^2 and *N6AMT1* rs2254638 polymorphisms on clopidogrel resistance in Chinese CAD patients. Both CYP2C19^*^2 and *N6AMT1* rs2254638 polymorphisms may serve as independent biomarkers to predict clopidogrel response in our population. Genetic testing especially for CYP2C19^*^2 is recommended when clopidogrel is prescribed for CAD patients in Chinese.

## Author contributions

All authors contributed to the study. X-PC designed the study and revised the manuscript, HL, Y-JZ, M-PL, X-LH, P-YS, and Q-LM performed the collection of subjects and genotyping, HL analyzed the data and wrote the manuscript, JT and WZ revised the manuscript.

### Conflict of interest statement

The authors declare that the research was conducted in the absence of any commercial or financial relationships that could be construed as a potential conflict of interest.
