# Supplementary material for: Association of N6AMT1 rs2254638 Polymorphism With Clopidogrel Response in Chinese Patients With Coronary Artery Disease
Source: Front Pharmacol. 2018 Sep 19;9:1039. doi: 10.3389/fphar.2018.01039 (PMC6156268; doi:10.3389/fphar.2018.01039)
Supplement: Supplementary file 1 [file Table_1.DOCX]

Supplement Table 1. PCR primers of 3 SNPs

| SNP | Forward Primer | Reverse Primer |
| --- | --- | --- |
| *CYP2C19*2* | 5’-tcagaggctgcttgatagaaatc-3’ | 5'-ccttgacctgttaaacatccgta-3' |
| *CYP2C19*3* | 5’- cttcaccctgtgatcccact-3’ | 5'-aaacatgccaattcagcaca-3' |
| *N6AMT1 rs2254638* | 5’-agagaagccacctggtgaga-3’ | 5’-ggtcccccaataatagagcagg-3’ |
