# Supplementary material for: Association of N6AMT1 rs2254638 Polymorphism With Clopidogrel Response in Chinese Patients With Coronary Artery Disease
Source: Front Pharmacol. 2018 Sep 19;9:1039. doi: 10.3389/fphar.2018.01039 (PMC6156268; doi:10.3389/fphar.2018.01039)
Supplement: Supplementary file 2 [file Table_2.DOCX]

Supplement Table 2. Genotype distribution of all the there SNPs in the entire cohort.

| SNP ID | Variation type | Location | MAF | *P* ^HWE*^ |
| --- | --- | --- | --- | --- |
| *CYP2C19*2* (rs42442850 | G>A | Chr10:94781859 | 0.221 | *0.520* |
| *CYP2C19*3* (rs4986893) | G>A | Chr10:94780653 | 0.014 | *0.835* |
| *N6AMT1* rs2254638 | T>C | Chr21:28883961 | 0.442 | *0.299* |
